# Using Wind Setdown and Storm Surge on Lake Erie to Calibrate the Air-Sea Drag Coefficient

**DOI:** 10.1371/journal.pone.0072510

**Published:** 2013-08-19

**Authors:** Carl Drews

**Affiliations:** NCAR Earth System Laboratory, National Center for Atmospheric Research, Boulder, Colorado, United States of America; Plymouth University, United Kingdom

## Abstract

The air-sea drag coefficient controls the transfer of momentum from wind to water. In modeling storm surge, this coefficient is a crucial parameter for estimating the surge height. This study uses two strong wind events on Lake Erie to calibrate the drag coefficient using the Coupled Ocean Atmosphere Wave Sediment Transport (COAWST) modeling system and the the Regional Ocean Modeling System (ROMS). Simulated waves are generated on the lake with Simulating WAves Nearshore (SWAN). Wind setdown provides the opportunity to eliminate wave setup as a contributing factor, since waves are minimal at the upwind shore. The study finds that model results significantly underestimate wind setdown and storm surge when a typical open-ocean formulation without waves is used for the drag coefficient. The contribution of waves to wind setdown and storm surge is 34.7%. Scattered lake ice also increases the effective drag coefficient by a factor of 1.1.

## Introduction

Strong winds acting on a body of water for a sustained number of hours may produce currents and a shift in the entire water mass. Wind setdown occurs when the body of water recedes from the upwind shoreline; storm surge occurs when the water shifts toward the downwind shoreline and inundates dry land. These two phenomena are opposite in vertical direction and comparable in magnitude; wind setdown is a drop in the water level, and storm surge is a rise in water level. This study analyzes the two effects acting on an enclosed body of water (Lake Erie), and compares them with observations in order to calibrate the parameters of a regional ocean model coupled with a wave model.

### 1.1 Prior Research

In 1969 Norman S. Heaps published a study of a two-dimensional model of storm surge on the North Sea.[1] He used a rectangular model grid oriented on latitude and longitude lines, with the open-sea and coastal boundaries of northwestern Europe. His grid resolution was about 34 km per grid cell, and his calculating time step was 6 and 3 minutes. Heaps wrote an ALGOL program to solve the finite-difference equations in 32 K of core memory. Wind forcing (speed and direction) at his grid points produced changes in the free surface *zeta* and in the u- and v-components of the mean current.

Despite the computational limitations present in 1969, Heaps was able to reproduce with remarkable accuracy the magnitude and timing of a "Hamburg surge" that occurred during a windstorm in February 1962. The North Sea rose 3.35 m at Cruxhaven, at the mouth of the Elbe estuary, with severe flooding in Hamburg. His model results show that he was unable to reproduce the higher-frequency oscillations present in observations taken at tidal measuring stations along the coasts. These oscillations were probably either reflections of surge off coastal features that his model grid could not resolve, or variations in the wind vectors that were not captured by the temporal resolution of his meteorological analysis (2 hours).

Modern oceanography recognizes the influence of spatial and temporal resolution on the accuracy of storm surge forecasts. Although ROMS uses the same gridded approach as Heaps used, for the North Sea I would increase the horizontal resolution by a factor of 10 and reduce the calculating time step to 1 minute. A modern surge model would also require a wind forcing field sampled at 30-minute intervals or smaller.

Heaps published a three-dimensional modeling study of the Irish Sea in 1973.[2] His grid resolution was 14 km, and his time step for calculation was 2 minutes. His numerical model used four vertical levels. Heaps considered his 3-D model to be an improvement over the earlier 2-D study because he could determine the vertical structure of the currents, instead of just the depth-averaged value. After several hours of wind stress there develops in the deep Irish Channel a strong wind-driven surface current and a corresponding return current at a depth of about 60 m. In an operational forecasting system it would be useful to report to ship captains the surface current, since it differs in speed and direction from the depth-averaged total current. Heaps found the greatest surge to occur at Solway Firth and Morecambe Bay, again matching local observations.

Neither the 1969 nor the 1973 studies included tides or barometric forcing. Surge modeling studies since then have taken advantage of greater computational power and disk storage to increase their spatial and temporal grid resolution. Coastal ocean modelers can now represent the nonlinear interactions between wind, tides, and barometric pressure. The SWAN wave model provides a way to calculate wave height and direction, thereby providing another valuable metric for navigation on open seas.

### 1.2 Drag Coefficient

The drag coefficient C_d_ determines the transfer of momentum between wind and the water surface. The drag coefficient is not constant, but depends on factors such as the wind speed, wave height and direction, and air temperature. A correct numerical formulation is crucial for accurately modeling storm surge. The wind stress τ upon the water surface is calculated as the product of the drag coefficient C_d_, the density of air ρ, and the square of the wind velocity measured at 10 meters above the surface:
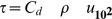
(1)


This study uses the Large & Pond formulation for the drag coefficient,[3] with a maximum value reached at 25 m/s according to Weisberg & Zheng.[4]

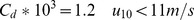
(2)

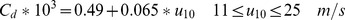
(3)

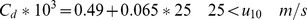
(4)


Note that the Large & Pond formulation is derived from open ocean conditions, whereas storm surge and wind setdown occur primarily in coastal areas. Several authors describe reductions made to the drag coefficient to account for higher wind speeds measured in hurricanes and typhoons.[5] [6] [7] This study compares Weisberg & Zheng (2006) to Oey et al. (2006) at the end of section 3.

### 1.3 Windstorms on Lake Erie

**Figure 1:**
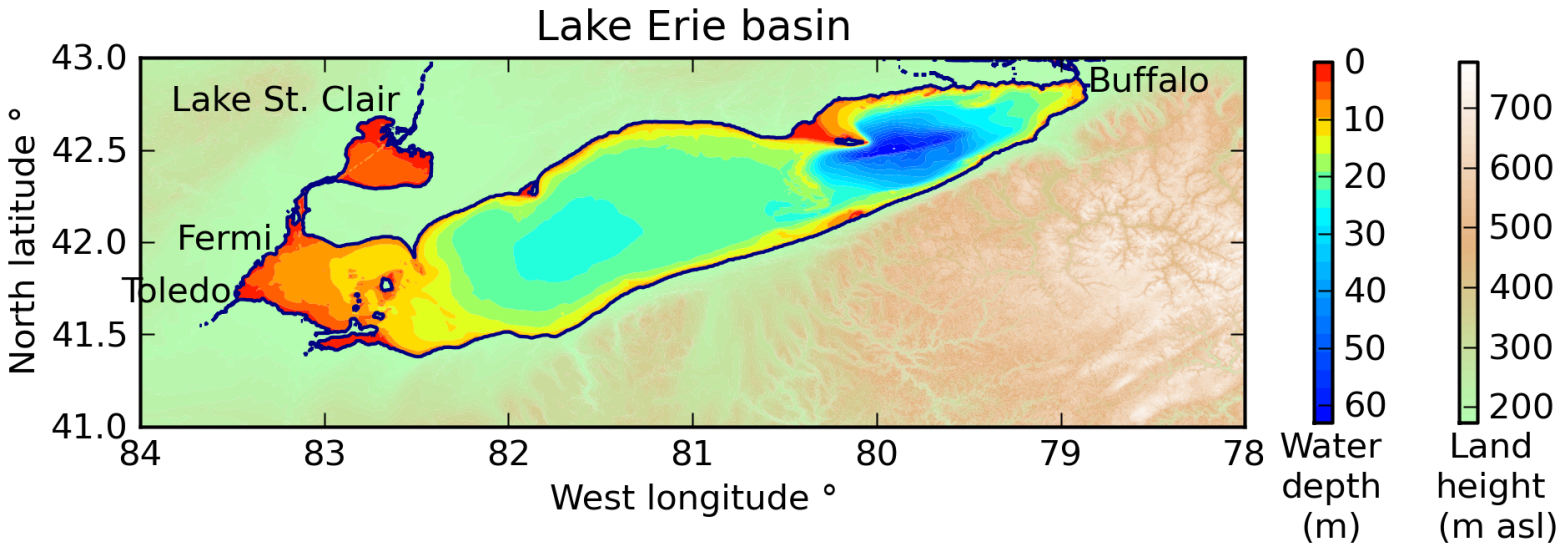
Topography and bathymetry of the Lake Erie basin. The lake is characterized by three major basins from west to east that are distinguished by their depth. There are NOAA measuring stations at the Fermi power plant, Toledo, and Buffalo.

Lake Erie is a large inland lake in North America, located between the United States and Canada. The lake is divided into three major basins, distinguished by their depth (see Figure 1). At an average depth of 19 m Lake Erie is the most shallow of all the American Great Lakes, and consequently it is susceptible to storm surge during high winds.[8] Since there are measuring stations around the lake's perimeter,[9] Lake Erie provides a useful test bed for hindcasting and validating model results. The primary axis of Lake Erie is aligned 20° north of due east. The lake is 400 km long in the west-east direction from Toledo, Ohio, to Buffalo, New York; and 90 km wide at its widest point from Ashtabula, Ohio, to Port Stanley, Ontario.[10].

On December 1–2, 2006 and January 30–31, 2008 there were strong windstorms over the Great Lakes that caused extreme surge events on Lake Erie. In both cases the wind came from the west, producing displacements between the water levels at the western and eastern ends of the lake of 4.2 m in 2006 and 5.1 m in 2008.[9] Ice was present on the lake on January 28, 2008, but was partially broken up by the windstorm.[11] This study analyzes these events and uses a regional ocean model to reproduce the water levels measured at both ends of the lake. The goal is to verify that the ocean model and drag coefficient produce accurate results, or to provide a numerical adjustment factor if the model results depart significantly from observed water levels.

### 1.4 Ocean and Wave Models

The Coupled Ocean–Atmosphere–Wave–Sediment Transport (COAWST) modeling system is an aggregation of ocean and atmospheric models that are coupled together for the purpose of examining coastal processes at regional scales.[12] COAWST uses the Model Coupling Toolkit to exchange data fields during a simulation run between the ocean model ROMS, the atmosphere model WRF (Weather Research and Forecasting),[13] the wave model SWAN, and the Community Sediment Transport Model.[14] Neither WRF nor the sediment model were used in this study. The COAWST modeling system may be obtained by contacting the United States Geological Survey (USGS) Woods Hole Science Center.[15].

The Regional Ocean Modeling System (ROMS) is an ocean general-circulation model suitable for modeling ocean behavior from local scale to basin scale. ROMS computes the hydrostatic primitive equations for momentum using a free surface and terrain-following coordinates in the vertical dimension. Shchepetkin & McWilliams have described the model numerics.[16] ROMS has been applied to ocean circulation in a variety of different regions, including shallow coastal estuaries.[17] [18] The wind-driven calculations have been compared favorably with observations of storm surge.[19] ROMS implements an algorithm for drying and wetting whereby the water's edge can recede and expose the underlying bathymetry, or advance to cover formerly dry land.

Wang et al. applied ROMS to modeling storm surge in the seas around Ireland.[19] They used the air-ocean drag coefficient described in Smith,[20] but did not incorporate waves into their calculations. Their results show that ROMS is capable of modeling individual surge events as well as long-term surge statistics with reasonable accuracy (∼10 cm). They did note a consistent **under**estimation of extreme surge events. For example, ROMS underestimated the surge event of 22–23 January 2002 by 20 cm at St. Mary's and Newlyn stations; the modeled surge height (45 cm) was 69% of the observed surge (65 cm). Furthermore, within the semi-enclosed Irish Sea ROMS produces a systematic **under**estimation of surge amplitude for almost all surge events, especially at the Bangor station.

SWAN (Simulating WAves Nearshore) is a wave model that computes random, short-crested wind-generated waves in coastal regions and inland waters.[21] ROMS and SWAN may be run in a coupled mode using the Model Coupling Toolkit (MCT).[22] SWAN accepts a wind forcing field to generate waves from a state of no motion.

In a Master's thesis, Weaver used ADCIRC (the ADvanced CIRCulation model for coasts, shelves, and estuaries) with SWAN-generated waves to evaluate the contribution of waves to storm surge.[23] In a hindcast simulation of Hurricane Georges (1998), he found that wave forcing contributes 25–33% of the total rise in water level.

## Methods

I created the ROMS domain by downloading gridded topography of the Lake Erie basin and converting it into ROMS format. I derived a wind forcing field from NOAA data products and applied it to the domain. The ROMS model parameters reflect fresh-water conditions of the lake during winter. The goal was to determine model parameters such that the model's output would match observed measurements of wind setdown and storm surge. Several sets of simulation experiments were run to analyze the effect of changing various model parameters. This section describes these stages in greater detail.

### 2.1 Bathymetry of Lake Erie

The National Geophysical Data Center supplied gridded bathymetry and topography of the Lake Erie basin via their GEODAS Grid Translator.[24] The grid resolution is 30 arc-seconds, which corresponds to 689 meters per grid cell in the x-direction (west-east) at the latitude of Lake Erie, and 926 meters in the y-direction (south-north). Table 1 lists the grid sizes for this domain, and Figure 1 shows the lake's bathymetry. Toledo is at the western end of the lake, and Buffalo is at the eastern end. The Fermi Power Plant is located north of Toledo on a more exposed section of shoreline. The domain also includes Lake St. Clair. The average lake level for the two wind events is 174 meters above sea level.

**Table 1:** Dimensions of the Lake Erie domain.

Grid parameter	Value
X domain length	496 kilometers
X grid cells	720
X cell size	689 meters
Longitude bounds	84° west to 78° west
Y domain length	222 kilometers
Y grid cells	240
Y cell size	926 meters
Latitude bounds	41° north to 43° north

### 2.2 Wind Forcing

Three major factors affect the displacement of the water's free surface in a windstorm: wind stress, wave radiation stress, and wave setup. Wind stress is the horizontal force τ exerted by the wind on the lake surface.[25](p. 29) τ acts on the entire water surface. Wave radiation stress is the momentum transfer from wind-generated waves. Wave setup is the increase in water level at the downwind shoreline caused by breaking waves. The barometric air pressure also affects the lake level as high and low pressure weather systems move across the lake.

The Wang paper emphasizes the importance of adequate spatial and temporal resolution for the wind forcing fields.[19] For this study NOAA supplied the forcing fields for wind and barometric pressure via their Rapid Update Cycle (RUC 252).[26] RUC 252 provides the best combination of high spatial and temporary resolution for North America, covering the study period 2006-2008. The horizontal grid resolution is 20 km, and the updates occur every hour. (NOAA also supplies a 13-km Rapid Update Cycle, but the archive only extends back to April 20, 2008.) The RUC 252 data product is delivered in a Lambert conformal projection; I mapped the RUC grid onto a latitude-longitude grid at 20 km resolution, then used linear interpolation to increase the resolution of the grid to match the ∼1 km Lake Erie domain (see Table 1). ROMS automatically interpolates the 1-hour time resolution of the forcing wind fields onto the time step of the model. The RUC surface air pressure is converted to the Lake Erie domain using the same algorithm.

The mapping algorithm does not provide for wind gusts, but only converts the sustained winds at 10 m above the surface. The gusts are not included because the standard formula (1) relating wind speed to surface stress uses only U10, the sustained wind speed at 10 m above the water surface.

**Figure 2:**
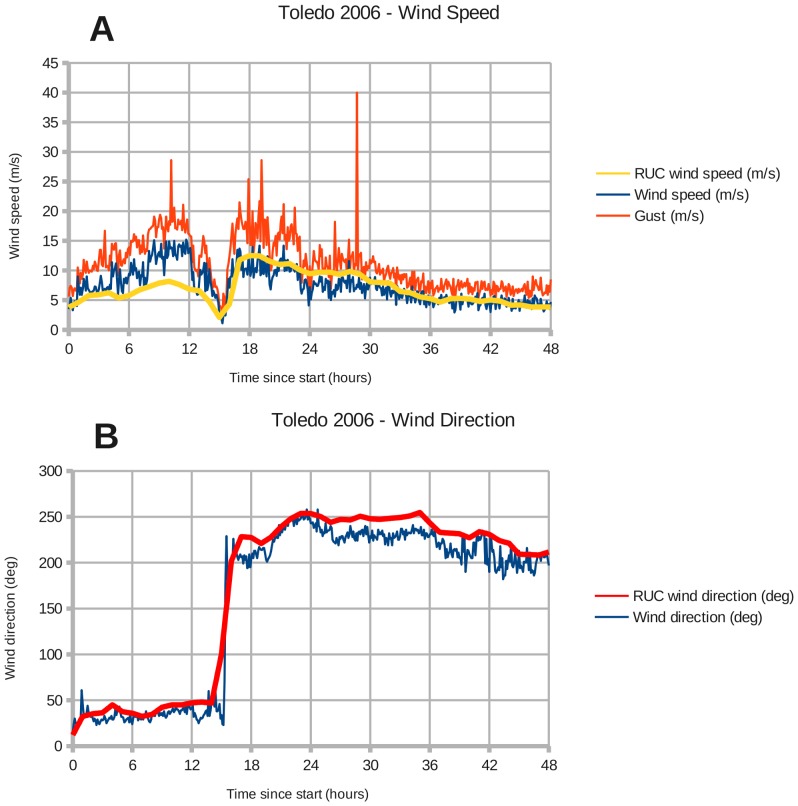
Wind forcing at Toledo during December 1–2, 2006. Panels: (A) Toledo wind speed, (B) Toledo wind direction. The lines labeled "RUC" represent wind data from NOAA's Rapid Update Cycle interpolated from their 20 km grid onto the 1 km Lake Erie domain (Section 2.2).

**Figure 3:**
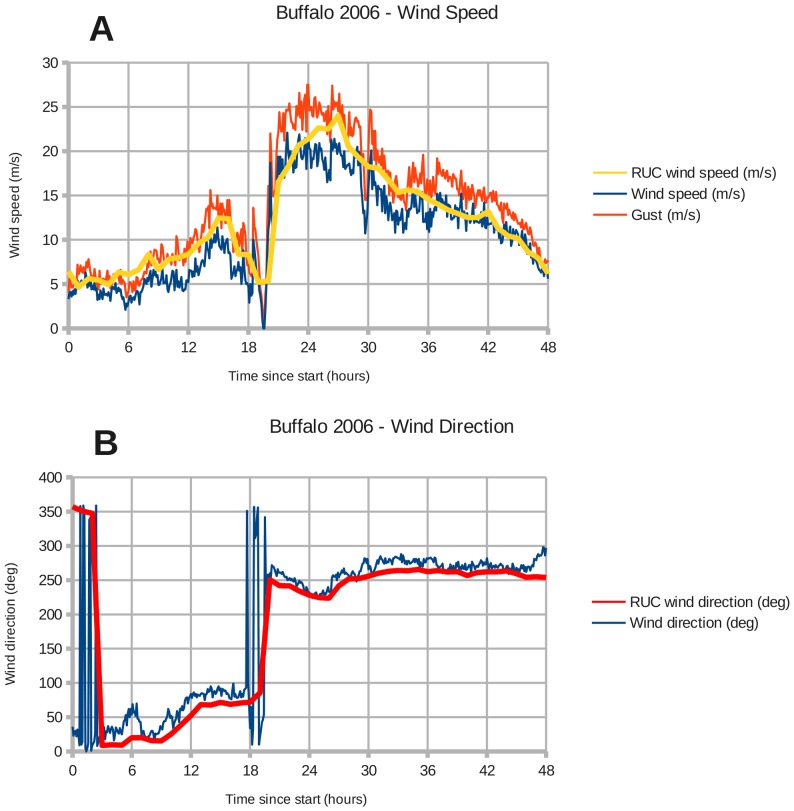
Wind forcing at Buffalo during December 1–2, 2006. Panels: (A) Buffalo wind speed, (B) Buffalo wind direction.

**Figure 4:**
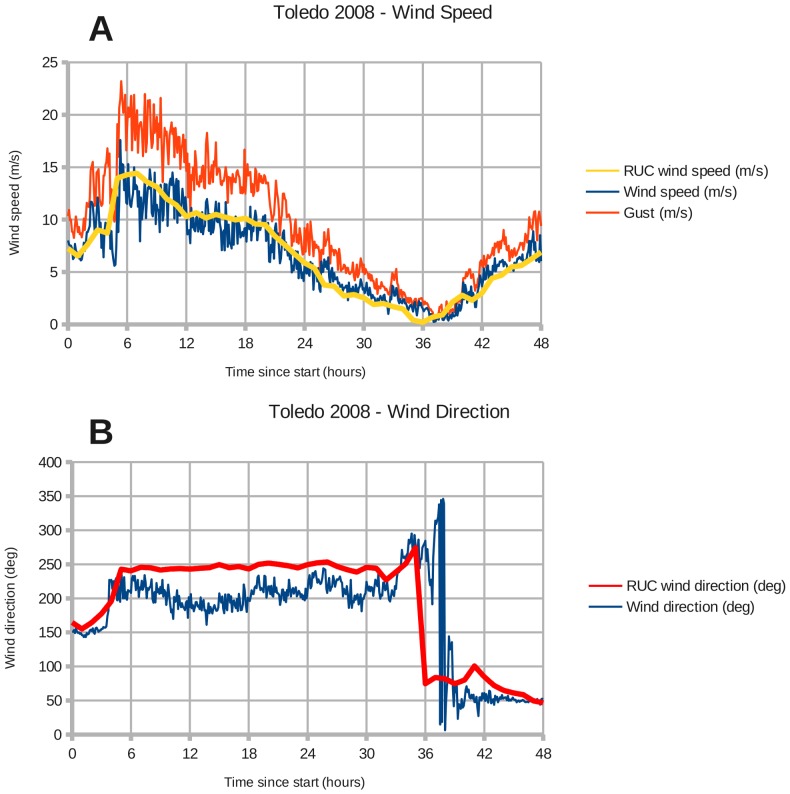
Wind forcing at Toledo during January 30–31, 2008. Panels: (A) Toledo wind speed, (B) Toledo wind direction. The lines labeled "RUC" represent wind data from NOAA's Rapid Update Cycle interpolated from their 20 km grid onto the 1 km Lake Erie domain (Section 2.2).

**Figure 5:**
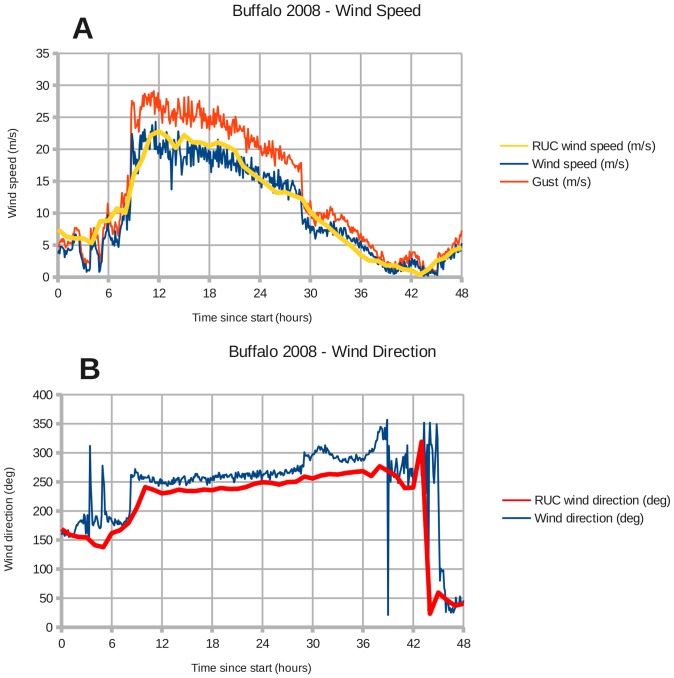
Wind forcing at Buffalo during January 30–31, 2008. Panels: (A) Buffalo wind speed, (D) Buffalo wind direction.

There are NOAA weather stations at Toledo and Buffalo that recorded the wind speed and direction during the two windstorms. Toledo station 9063085 is located at (41.6933 North, 83.4717 West), and Buffalo station 9063020 is located at (42.8767 North, 78.8900 West). Figures 2–5 show the modeled wind speed and direction versus the actual winds observed at Toledo and Buffalo for the two wind events. The 2008 event marked the passage of a cold front through the region. Although there are discrepancies between the RUC interpolated grid and station measurements, a visual inspection reveals no overall under- or over-estimation of the wind speed. The RUC grid, when interpolated from 20 km onto a 1 km grid, should provide sufficient accuracy for coarse validation of the drag coefficient in coastal areas. Fine-tuning will require higher-resolution wind fields.

### 2.3 ROMS and SWAN Model Parameters

In winter the water column of Lake Erie is well-mixed with a uniform potential temperature of 0–2 degrees Celsius.[27] Astronomical tides on the Great Lakes are on the order of 1–3 cm,[28] [29] [30] and were not modeled in this study. The salinity (0.0 PSU) and density (1000 kg/m^3^) parameters represent fresh water. Water temperature was 2° Celsius. Model experiments ran for 48 hours using a time step of 3 seconds, and they recorded data every 6 minutes. The 3-D configuration used 3 vertical levels. The vertical mixing scheme was Generic Length Scale (GLS_MIXING). Forcing by barometric pressure was turned on. Wetting and drying was turned on in order to model the change in shoreline with fluctuating water levels (WET_DRY). The critical depth was 0.3 m (the minimum depth of water for wet/dry).

Wave breaking surface flux from wave amplitude was activated (TKE_WAVEDISS). Wave dissipation from the SWAN wave model was activated (WDISS_WAVEMOD).

SWAN ran in two-dimensional mode with varying wind forcing (DYNAMIC). The baseline water level was 174.1 m above sea level in December 2006, and 173.9 m in January 2008, according to the NOAA measuring stations. Bottom friction was activated by the command FRICTION, and depth-induced breaking was activated by the command BREAKING. SWAN used a calculation time step of 3 minutes, and exchanged data with ROMS every 6 minutes.

The model versions were: ROMS/TOMS version 3.4, SVN Revision 663, SWAN 40.81.

### 2.4 Observed Setdown and Surge

The measuring station at Toledo (9063085) includes a gauge for measuring the water level. On 2 December 2006 GMT this station recorded a drop in the water level from 174.1 m to 172.35 m, or 1.75 m below the normal lake level on the previous day. On 30 January 2008 GMT this station recorded a drop in the water level from 173.9 m to 171.55 m, or 2.35 m below the normal lake level.[9] Unfortunately, the ship channel leading from the lake proper to the measuring station at Toledo is only 340 m wide, and therefore cannot be resolved using the ROMS grid resolution of 689 m in the x direction and 926 meters in the y direction. The resulting effect when attempting to model the Toledo station is that the station grid cell loses its connection to the rest of the lake as certain grid cells along the ship channel become dry, and the station cell ceases to be a reliable measure of wind setdown at the western end of the lake.

I chose to use the NOAA measuring station at the Fermi Power Plant for the water level instead of the Toledo station. Fermi station 9063090 is located at (41.9600 North, 83.2567 West), on an exposed section of shoreline 35 km northwest of Toledo that provides good fluid communication with the western basin of Lake Erie. The Fermi station does not record meteorological observations, only water level. The measuring station at Buffalo is located along a section of open waterfront that provides open access to the eastern basin of Lake Erie.

**Figure 6:**
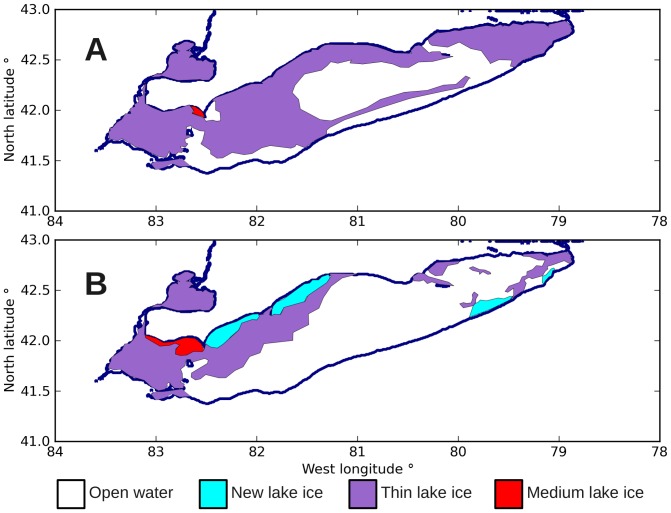
Ice coverage on Lake Erie, winter 2008. Panels: (A) 28 January 2008, (B) 31 January 2008. The windstorm cleared much of the ice from Lake Erie. These images were created using archive data from the Canadian Ice Service, Government of Canada.[11]

Figure 6 shows the ice coverage on Lake Erie during January 28 and 31, 2008. The windstorm appears to have blown the water surface partially clear. I expected that broken lake ice would increase the air-water drag coefficient by increasing the surface friction and providing a mechanical linkage for transferring surface momentum downward.

### 2.5 Experiments

**Figure 7:**
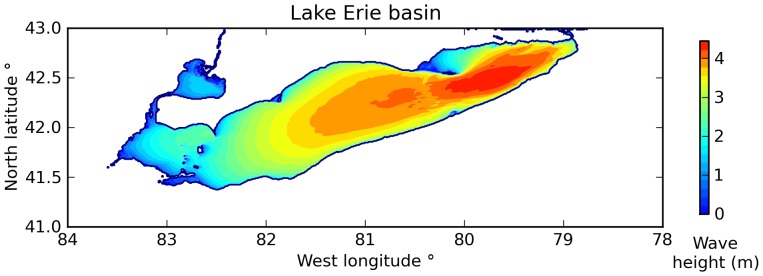
Significant wave height on Lake Erie in steady-state (experiment S1). SWAN generated these waves driven by winds at 20 m/s blowing from the southwest and aligned with the long axis of the lake (20° north of due east).

I first ran a "stationary" calculation S1 with SWAN to determine the magnitude of wave heights under strong winds (20 m/s) of constant speed and direction (Figure 7). This steady-state calculation established the size of waves that one should expect for the dynamic simulations. SWAN calculated a maximum significant wave height of **4.44 m** in the eastern basin of the lake. Although this wave height may seem extreme for an inland body of water, larger waves have been recorded. In "April 1979, the 315 ft ship *Labradoc*, with a crew of 20, foundered on Lake Erie in 20 ft seas and 45 kt winds. Fortunately all hands aboard *Labradoc* were rescued by helicopter."[31].

A second stationary calculation S2 established the relationship in SWAN between the wind speed and the calculated wave height on Lake Erie. Calculation S1 was repeated for wind speeds ranging from 0 to 30 m/s, and the maximum wave height on the lake was recorded. The resulting plot should match the theoretical result, that wave height is proportional to the square of the wind speed.[32](formula 16.35)

32 simulation experiments were performed among the December 2006 and January 2008 test cases. Only the significant experiments are described here; the remaining model runs represent incremental adjustments of model parameters. The general approach was to calibrate the model parameters first at Fermi in 2006, using the lack of breaking waves on the upwind side of the lake to eliminate wave setup as a variable. Then the calibration effort shifted to Buffalo in 2006, which presumably involved wave setup on the downwind shore. Finally the additional effect of lake ice was determined from the 2008 case.

Experiments E1-E4 represent several approaches to modeling the influence of waves on wind setdown and storm surge. E1 represents ROMS running in its two-dimensional mode, while E2 uses the three-dimensional mode with 3 vertical levels. Experiment E3 incorporates the Mellor (2008) method for calculating nearshore radiation stress,[33] using significant wave heights generated by SWAN from the forcing wind field. E4 uses the newer vortex force calculation for SWAN-generated waves (WEC_VF).[34] [35].

Experiments E16 and E17 explore the effect of the number of vertical levels in the ROMS ocean model. A future objective of this project is to prepare for an eventual forecasting system that can provide estimates of storm surge in faster than real time. The number of vertical levels affects the speed of the calculation. These two experiments are useful in considering a trade-off between calculation speed and model accuracy.

Experiment E18 adjusts the quadratic bottom drag coefficient (RDRG2) as a way of matching the timing of the ROMS model with observations.

Experiment E21 reduces the power of vortex force by 20% in order to reduce the model's overshoot and bring the ROMS results closer to observations in 2006. The vortex force calculation is not readily accessible to the COAWST user, so I reduced the wind speed passed into SWAN instead. Winds passed to SWAN were first multiplied by the square root of 0.8. Since wave heights are proportional to the square of the wind speed,[32](formula 16.35) the effect is to multiply the wave heights and vortex force by 0.8. Note that this theoretical relationship was verified by experiment S2.

Experiment E23 performs simple data assimilation in the eastern half of the Lake Erie domain in an attempt to match the RUC wind speeds with NOAA observations at Buffalo. The RUC wind speeds used to force ROMS were adjusted to eliminate the discrepancy shown in Figure 3, panel A, during the time interval from 24 to 36 hours.

Experiments E25, E26, and E31 apply to January 2008, when there was ice on Lake Erie. These experiments use different values of an ice adjustment factor to match the modeled wind setdown and storm surge with the measured water levels at Fermi and Buffalo. The ice adjustment factor is a number ranging from 1.0 to 1.3 that multiplies the air-water drag coefficient C_d_. I also multiplied the winds passed into SWAN by the square root of the same factor to achieve a corresponding increase in wave height due to increased wind stress.

Experiment E22 doubles the lake depth to determine the effect of water depth on wind setdown and storm surge. E22 compares to E21 from December 2006.

Experiments E28, E29, and E30 isolate the wave contribution to storm surge by reducing the significant wave heights to much smaller values. This reduction is accomplished by multiplying the wind speed passed to SWAN by the square root of 0.01 in E28, and by the square root of 0.1 in E29 and E30. In this way COAWST was configured for near-zero wave contribution.

Experiments E35 and E36 replace the air-sea drag coefficient described by Weisberg & Zheng[4] with the formulation derived by Oey et al.[6].

Experiments T21, T23, T24, and T25 apply the calibration values calculated in the present study to the Lake of Tanis research published in 2010.[18] For the Tanis case study, the model result of interest is not the change in water level, but the duration of the dry land bridge that forms across the Kedua Gap.

Model results are available for download at the Earth System Grid, and may be retrieved through the NCAR Gateway by registering there.[36].

## Results

**Figure 8:**
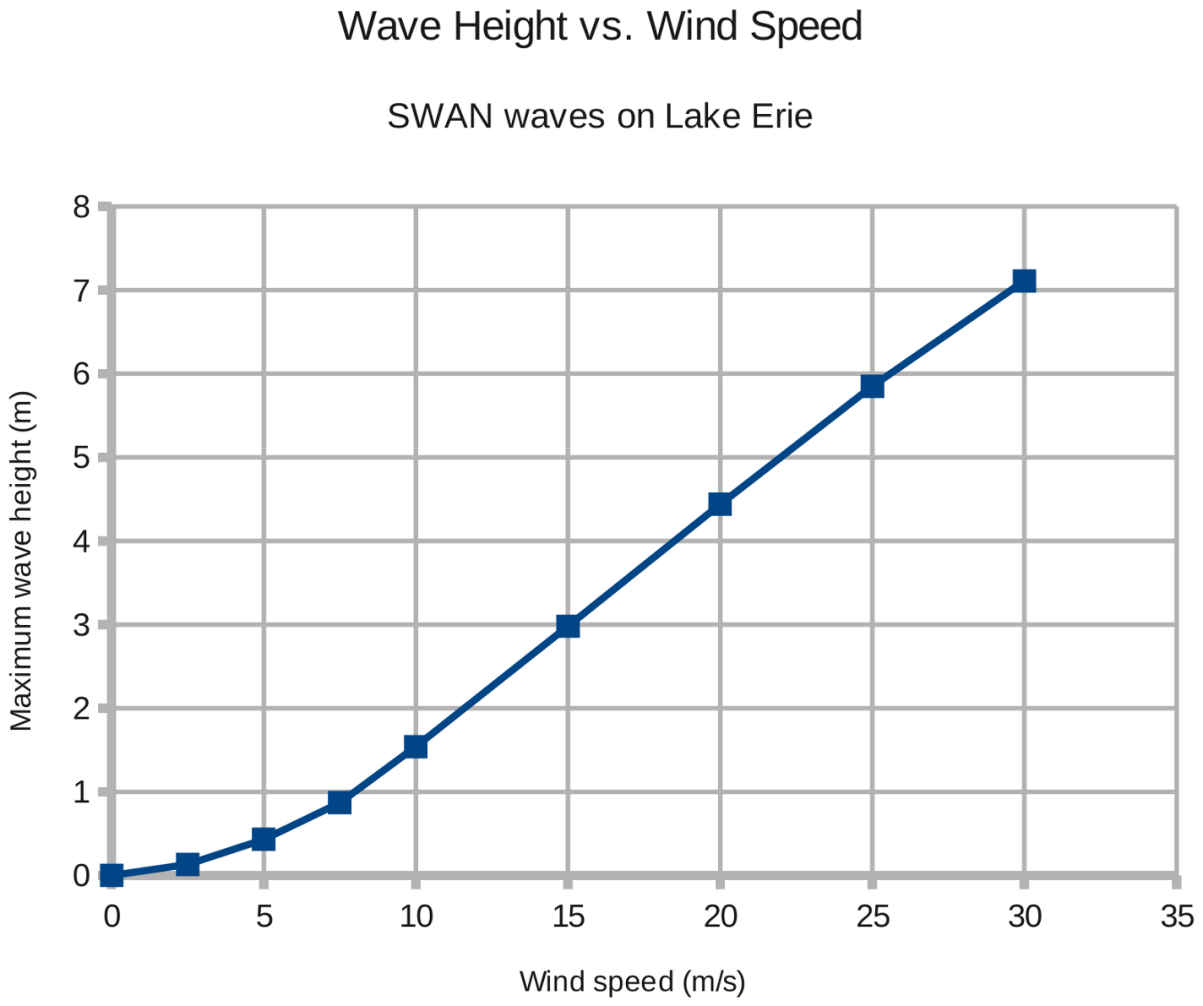
Maximum wave heights on Lake Erie for constant wind speeds (steady-state experiment S2). Wave height increases with the square of the wind speed until about 15 m/s, when wave growth is constrained by the lake's limited depth.

Experiment S2 displayed a relationship between wind speed and wave height that exhibits exponential behavior until about 15 m/s. At wind speeds greater than 15 m/s, the relationship becomes linear (Figure 8). The limited depth of Lake Erie begins to limit the maximum height of waves at wind speeds beyond 15 m/s. I used a log-log analysis to calculate the exponent within the curve segment from 2.5 to 15 m/s; the value is **1.75**. This exponent is close to the theoretical power of **2** by which wind speed relates to wave height.[32](formula 16.35).

The focus of the dynamic experiments was to match the greatest magnitude and timing of the observed setdown and surge events. Since an operational forecasting system would be concerned primarily with saving lives and property, the extreme events are the most significant for this study. Less attention was paid to the secondary oscillations in the water level.

Experiments E23 and E26 are "terminal experiments" in that they represent the final model configuration after the relevant parameters have been adjusted to match observations for December 2006 and January 2008. The largest waves on the lake in E23 were 4.2 m high; the largest waves in E26 were 4.8 m high. These results are close to the "stationary" wave height of 4.44 m calculated in experiment S1, indicating that the significant wave heights calculated by SWAN are consistent between the steady-state configuration and the experiments run with dynamic wind forcing.

**Figure 9:**
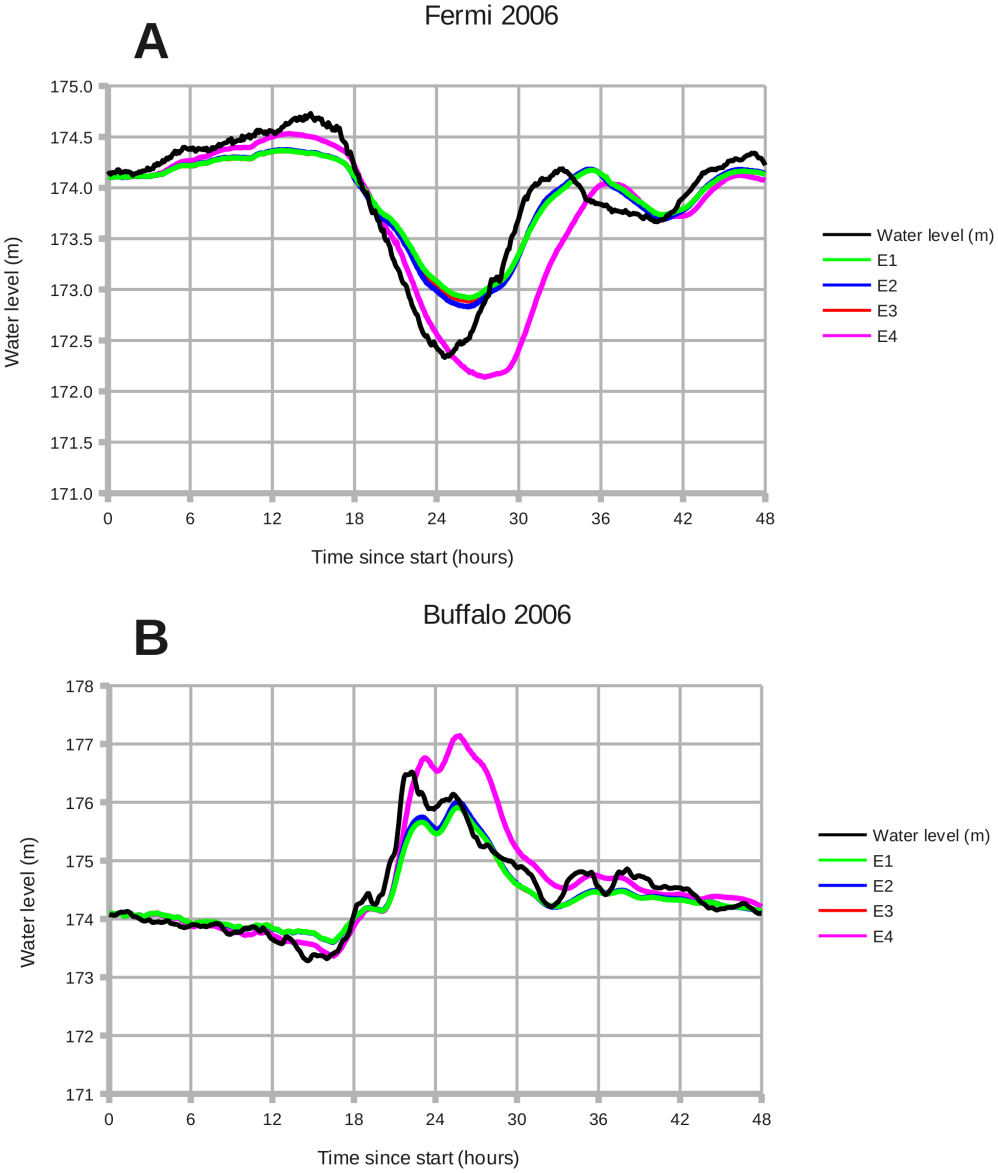
December 2006: Wind setdown and storm surge, and simulation experiments E1–E4. Panels: (A) Fermi 2006, (B) Buffalo 2006. **E1**: ROMS in 2-dimensional mode. **E2**: 3-D mode with 3 vertical levels. **E3**: SWAN-generated waves and nearshore radiation stress (NEARSHORE_MELLOR08).[33] **E4**: SWAN-generated waves and vortex force.[35] [34] Line E3 lies between E1 and E2. Vortex force causes waves to have a larger effect than other the methods.

Figure 9 shows the results of simulation experiments E1 – E4. Running ROMS in two-dimensional or 3-D mode causes only a small change in the water level. The Mellor (2008) algorithm for calculating wave radiation stress[33] produces no significant contribution from waves. The vortex force method[34] [35] causes waves to have a much greater effect; in fact, vortex force produces an **overshoot** in the water levels observed during wind setdown and storm surge.

**Figure 10:**
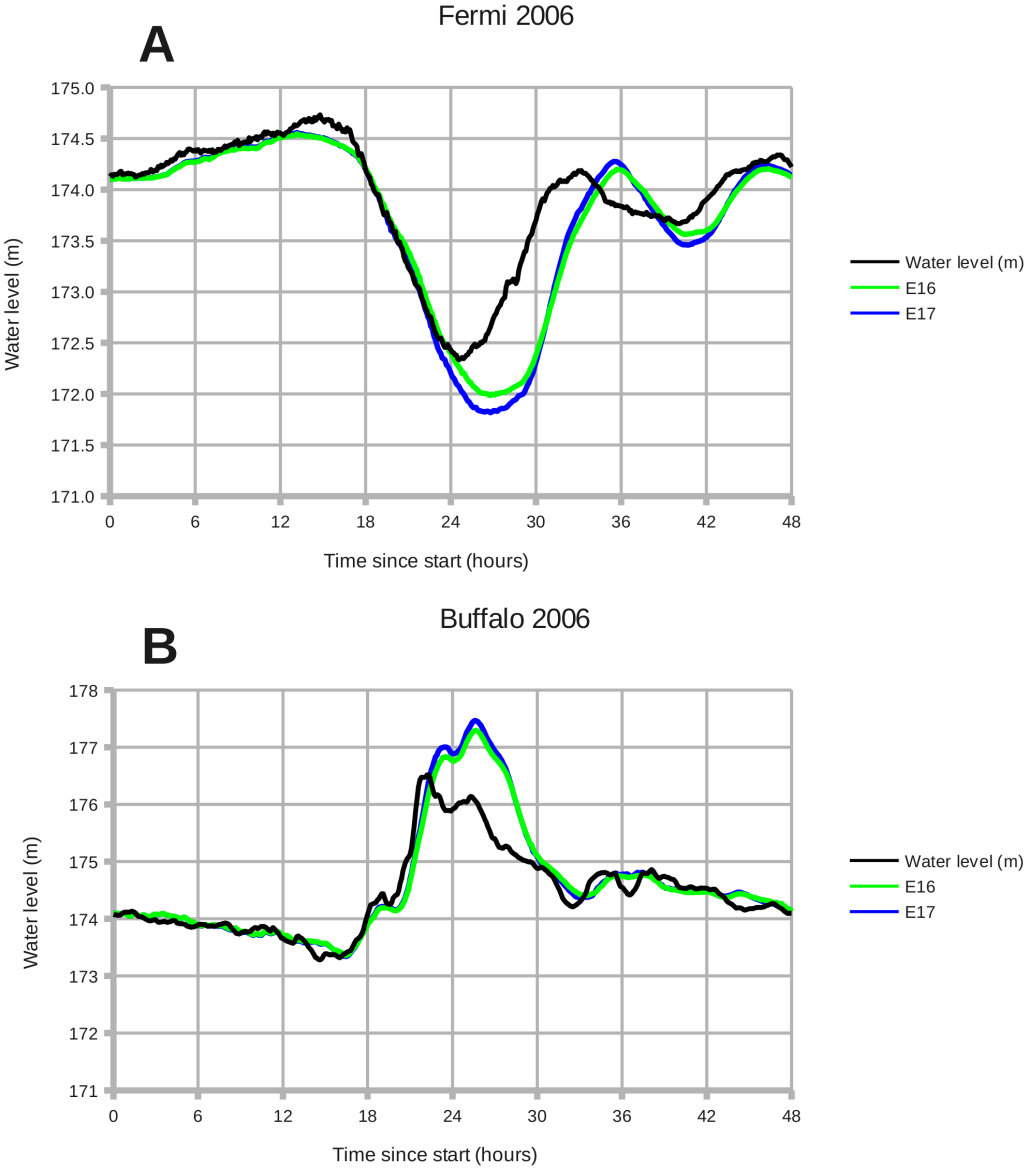
December 2006: Wind setdown and storm surge, with experiments E16–E17. Panels: (A) Fermi 2006, (B) Buffalo 2006. **E16**: 3 vertical levels. **E17**: 10 vertical levels. Increasing the number of vertical levels from 3 to 10 causes only a small increase in wind setdown and storm surge for this case.

Figure 10 shows the results of simulation experiments E16 and E17. Increasing the number of vertical levels in the ROMS ocean model from 3 to 10 changes the water level at Fermi and Buffalo by 17 cm; this change represents 7% of the setdown at Fermi and 5% of the surge at Buffalo calculated with 10 levels. Experiment E16 took 36.5 hours to run on a Dell Optiplex 960 workstation, while experiment E17 took 80.5 hours to run. The Dell workstation has an Intel Core 2 Quad processor running at 2833 MHZ.

**Figure 11:**
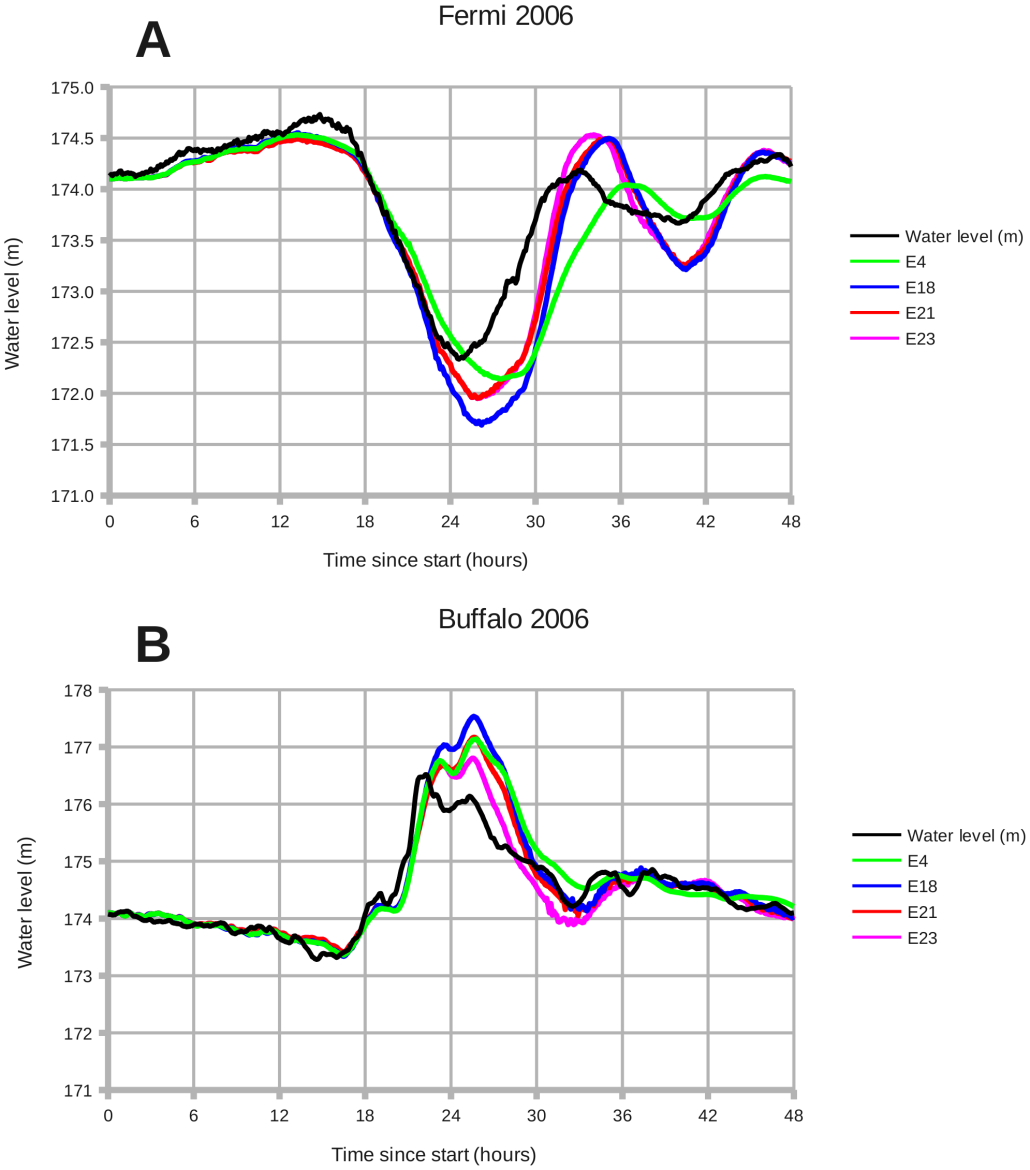
December 2006: Wind setdown and storm surge, with experiments E4, E18, E21, and E23. Panels: (A) Fermi 2006, (B) Buffalo 2006. **E4**: RDRG2  =  3.0e-3. **E18**: RDRG2  =  1.0e-3. **E21**: Vortex force * 0.8. **E23**: Adjusted the modeled wind speed at Buffalo. Decreasing the bottom drag RDRG2 improves the timing of the model by shortening the lake's reaction time to changes in wind stress (E4 -> E18). Decreasing the vortex force corrects the overshoot of setdown and surge (E18 –> E21). Simple data assimilation at Buffalo provides a further small improvement (E21 –> E23).

Figure 11 shows a time series of water levels for experiments E4, E18, E21, and E23. Between experiments E4 and E18 the quadratic bottom drag coefficient (RDRG2) was reduced from 3.0e-3 to 1.0e-3. This change causes the simulated lake to react quicker to wind stress. In panel A, the timing of line E18 is closer than E4 to observations during the interval from 24 to 48 hours. In panel B the line representing E18 decreases from the maximum surge quicker than E4 does (near 30 hours). Lake E4 is "too sluggish". Unfortunately, decreasing the value of RDRG2 also increases the overshoot; that is, the error in magnitude between model results and observations. The following experiments seek to rectify this problem.

Experiment E21 in Figure 11 represents a reduction in the vortex force by 20%. This reduction brings the peak setdown and surge values closer to the observed water levels. Although a further reduction continues to narrow the difference, I am reluctant to suggest a larger adjustment before the ocean modeling community has had a chance to test vortex force in a variety of coastal situations.

Experiment E23 in Figure 11 shows an improvement in the ROMS model results during the time interval from 24 to 36 hours. Since the data assimilation was carried out only in the eastern half of the Lake Erie domain, the improvement is consequently greater in panel B, which represents Buffalo. The secondary peak at Buffalo is smaller than E21 and closer to the observed water level.

**Figure 12:**
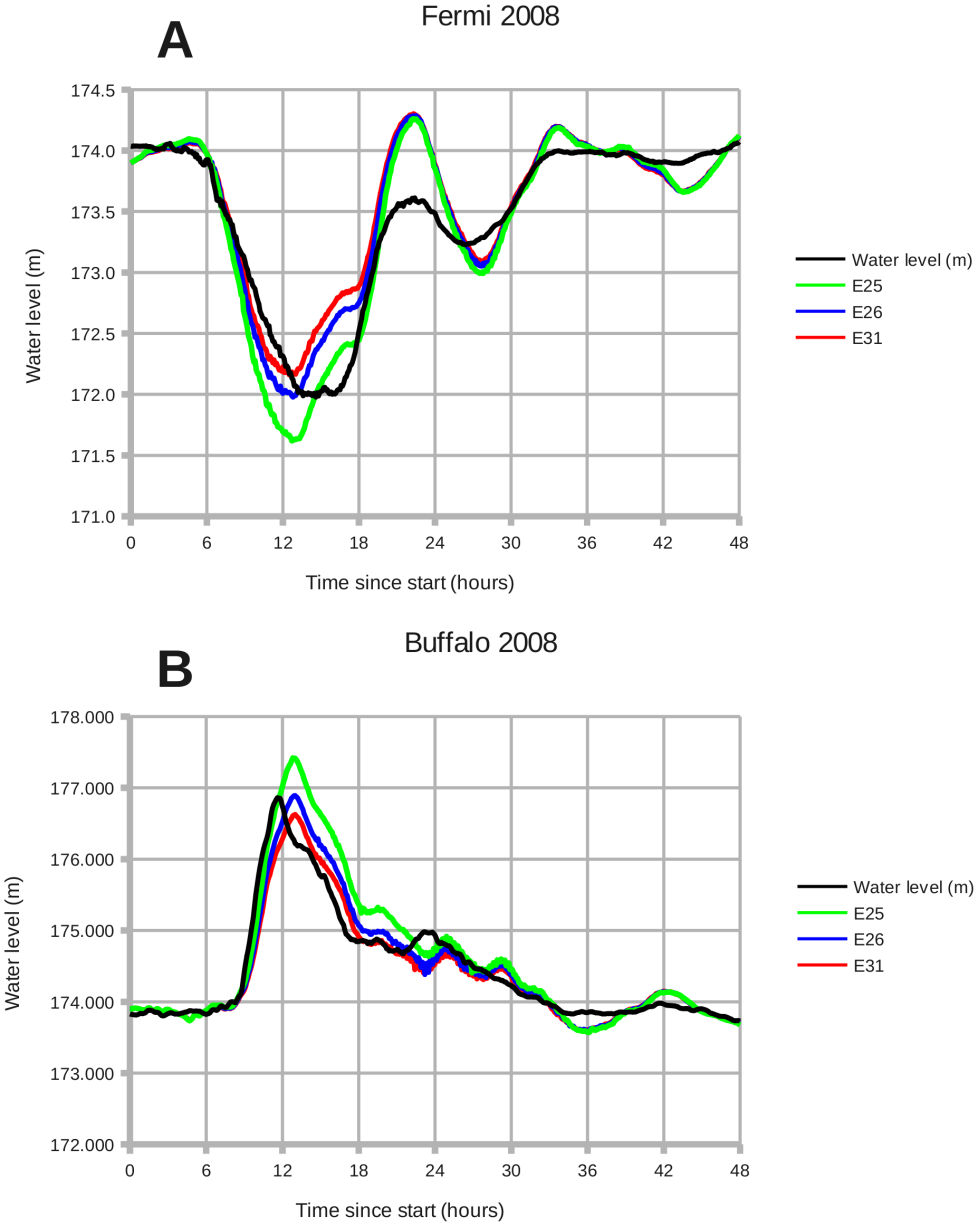
January 2008: Wind setdown and storm surge, with experiments E25, E26 and E31. Panels: (A) Fermi 2008, (B) Buffalo 2008. **E25**: Drag coefficient C_d_ * 1.3. **E26**: C_d_ * 1.1. **E31**: C_d_ * 1.0. Lake ice increased the effective drag coefficient by a factor of 1.1.

Figure 12 shows simulation experiments E25, E26, and E31 for January 2008. Experiment E31 represents the adjustments from December 2006 applied to 2008; the modeled setdown and surge falls short of observations. Experiment E25 uses an ice adjustment factor of 1.3, causing the ROMS model results to overshoot the observed water levels. Experiment E26 uses an ice adjustment factor of 1.1 and achieves a good match for the peak setdown and surge.

Doubling the lake depth in experiment E22 causes a reduction in the maximum wind setdown at Fermi from –2.15 m to –1.00 m, or 46.5% of the original change in water level. E22 produces a reduction in the peak storm surge at Buffalo from 3.05 m to 1.48 m, or 48.5% of the original displacement. Thus experiment E22 confirms the theoretical and observational finding: **shallow water is more susceptible to storm surge.** The shallow bathymetry of Lake Erie is the primary reason why it experiences greater storm surge than the other Great Lakes.

Experiment E28 reduces the SWAN wind by a factor of 100, producing no waves at all. Simulation experiment E29 in December 2006 reduces the wind forcing to SWAN by a factor of 10, producing a maximum wave height of 0.14 m. The change in water level is –1.47 m at Fermi, and 1.83 m at Buffalo. These results are identical to E28. Experiment E30 in January 2008 uses the same reduction by a factor of 10. The change in water level is –1.20 m at Fermi and 1.87 m at Buffalo.

By comparing experiment E29 with E23, and E30 with E26, we may obtain the final wave contribution to wind setdown and storm surge. These results are summarized in Table 2. For December 2006: the wave contribution to setdown is 31.6%, and the wave contribution to surge is 32.2%, for an average wave contribution to vertical displacement of 31.9%. For January 2008: the wave contribution to setdown is 37.5%, and the wave contribution to surge is 37.5%, for an average wave contribution to vertical displacement of 37.5%. Over both experiments, the average wave contribution to the vertical displacement in water level is **34.7%**.

**Table 2:** Modeled wind setdown and storm surge (m).

December 2006	Fermi	Buffalo
E23	–2.15	2.70
E29	–1.47	1.83
**January 2008**		
E26	–1.92	2.99
E30	–1.20	1.87

Experiments E29 and E30 represent negligible wave heights; the difference between them and experiments E23 and E26 is the wave contribution.

These results compare favorably with Wang et al.[19] Their underestimation of the storm surge event during 22–23 January 2002, when calculating without waves, produced a modeled surge that was 69% of the observed surge (Section 1). This result implies a wave contribution of 31%, which is close to the average value calculated here of 34.7%.

This wave contribution of 34.7% is slightly larger than the maximum contribution calculated by Weaver, which was 33%.[23] The coupled combination of COAWST-ROMS-SWAN produces results that are close to the ADCIRC-SWAN modeling system used by Weaver.

The drag coefficient formulated by Oey et al.[6] provides a smaller value for *C_d_* than Weisberg & Zheng[4] for wind speeds greater than 20 m/s. Since the combination of Weisberg & Zheng (2006) with vortex force **exceeds** the observed wind setdown and storm surge, experiments E35 and E36 use Oey (2006) instead to **reduce** this overshoot. Table 3 compares the two formulations for the drag coefficient *C_d_*. Oey 2006 reduces the overall average error from +8.5% to +4.8%.

**Table 3:** Comparison of drag coefficients.

**2006**	**Observed**	**E23**	**Percent**	**E35**	**Percent**
Fermi	172.33	171.95	+21%	172.00	+19%
Buffalo	176.52	176.80	+12%	176.65	+5.4%
**2008**	**Observed**	**E26**	**Percent**	**E36**	**Percent**
Fermi	171.98	171.98	0%	172.00	–1.1%
Buffalo	176.86	176.89	+1%	176.75	–3.9%

Experiments E23 and E26 use Weisberg & Zheng (2006).[4] Experiments E35 and E36 use Oey et al. (2006)[6] The lake levels are measured in m above sea level. The "Percent" column shows the error between model results and observations as a signed percentage of the vertical displacement. The percentages are signed according to overshooting the observed change in water level (+) or underestimating the observed change (–).

## Discussion

Lake Erie is a valuable natural laboratory, with weather stations and water level gauges around the lake. Wind setdown at the western end of the lake provides an opportunity to calibrate the air-water drag coefficient without the added complication of incoming waves breaking upon the measured shoreline. The wind events of December 2006 and January 2008, plus any other storms that may occur, are useful in matching ocean model results with observations.

### 4.1 An Operational Forecasting System

A high-resolution wind field is important for modeling the lake's water level. The experiments would likely be improved by employing a wind forcing field with greater spatial resolution. The RUC 252 data product used here must be interpolated from 20 km to match the 808 m average grid cell size in the Lake Erie domain, and that interpolation cannot capture the finer wind features that occur on length scales smaller than 20 km. Nevertheless, the intent of this paper is to provide the scientific basis for an **operational** forecasting system; wind fields on the order of 1 km horizontal resolution may not be available in real-time in a production environment. The RUC 252 product from NOAA represents a realistic and readily available source of wind data.

In an operational environment, where calculation time must be reduced to a minimum, it may be practical to run the ROMS ocean model in a configuration that would not be advisable in a research environment. Decreasing the number of vertical levels sharply reduces the computational cost of a model run. Experiments E16 and E17 in Figure 10 show that running the model with 3 vertical levels instead of 10 has only a small effect on the modeled water levels. Yet calculating with 3 vertical levels takes only 45% of the 10-level calculation time. Cutting the calculation time in half may justify the small loss in accuracy here if the forecasting timeline is short. These results are important to anyone interested in configuring a production system for storm surge forecasts.

Experiment E1 runs the Lake Erie surge model in the ROMS two-dimensional configuration, effectively reducing the number of vertical levels to 1. Figure 9 shows the comparison between E1 and the ROMS three-dimensional configuration E2. The 3-D configuration increases the setdown magnitude by 7.6% at Fermi and the surge magnitude by 5.5% at Buffalo. Thus there is only a small difference between 2-D and 3-D operation when barometric forcing and wave effects are omitted.

The three-dimensional configuration of ROMS / COAWST does not support barometric forcing or the vortex force algorithm. As Figure 9 illustrates, these two effects are necessary to bring the ocean model results closer to observations. ROMS is designed to be run as a three-dimensional model.

It was awkward to manipulate the significant wave height indirectly using the square of the wind speed passed to SWAN. Nevertheless, this approach appears to be the only way for a model user to adjust the vortex force without modifying the FORTRAN code directly. I expect that the manipulation of SWAN wind speed will become unnecessary as further improvements are made to the vortex force algorithm.

### 4.2 Application

Drews and Han published model results of wind setdown at the Kedua Gap, a reconstructed body of water in the eastern Nile delta circa 1250 BC.[18] That study reported the emergence of a 4-hour dry crossing on a land bridge at Tell Kedua that forms under wind stress. Since Drews and Han (2010) made no provision for waves in their ocean model, and since they used a value for quadratic bottom drag coefficient (RDRG2) of 3.0e-3, the present study provides an opportunity to apply the correction factors recommended here to see how they would affect those earlier results.

**Figure 13:**
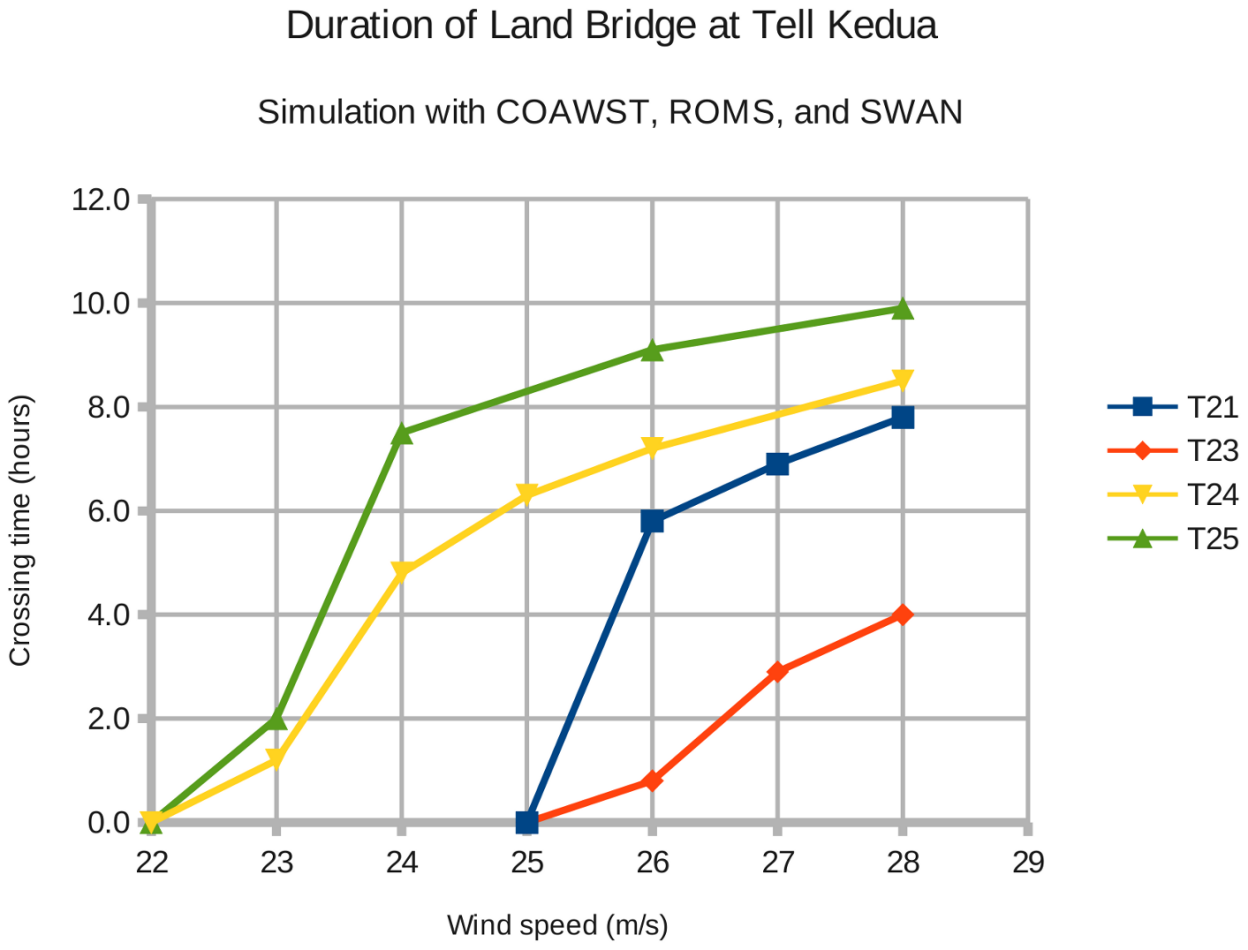
Corrections applied to the Lake of Tanis and the Kedua Gap. Reducing the quadratic bottom drag increases the duration of the dry land bridge, as does the inclusion of waves in the ocean model. **T23**: original configuration using RDRG2  =  3.0e-3 and no waves. **T21**: RDRG2  =  1.0e-3 and no waves. **T24**: RDRG2 =  3.0e−3 and SWAN-generated waves. **T25**: RDRG2  =  1.0e−3 and SWAN waves.

Drews and Han (2010) were primarily interested in the **duration** of the dry passage at Kedua, not in the **vertical change** in water level during wind setdown and storm surge. Consequently, the new simulation experiments performed here also focus on the duration of the dry crossing. The two corrections (RDRG2 and vortex force) were applied separately and in combination. The baseline wind speed is 28 m/s; Figure 13 shows the results for other wind speeds. The coastal configuration is Tanis experiment T14.

Simulation experiment T23 represents the original baseline configuration from 2010.[18] T23 uses RDRG2  =  3.0e−3 and no SWAN-generated waves. The dry crossing is open for **4.0 hours** under a wind speed of 28 m/s. Tanis experiment T21 reduces RDRG2 from 3.0e−03 to 1.0e−03; the dry passage opens at 5:30 hours and closes at 13:18, for a total crossing time of **7.8 hours**. The Lake of Tanis reacts quicker to wind stress, just as Lake Erie did. T24 uses RDRG2  =  3.0e−3 and adds waves with vortex force; the crossing time is **8.5 hours**. T25 uses RDRG2  =  1.0e−3 and SWAN-generated waves; the land bridge remains open for **9.9 hours**.

Figure 13 summarizes these model results graphically. The corrections to the quadratic bottom drag coefficient and the addition of SWAN waves more than double the duration of the dry passage at the Kedua Gap for winds at at 28 m/s. For a passage time of 4.0 hours, the wind speed required is now 24 m/s.

When the duration of the dry land bridge falls to 0.0 hours with decreasing wind speed, it indicates that there is a **balance** between the wind stress and the fluid pressure exerted by the body of water pushed back by the wind. Since the upper and lower pairs of curves in Figure 13 "pinch off" and converge at 0.0 hours, it shows that this force balance is not affected by changes in the bottom drag RDRG2. The bottom drag coefficient only affects the **time** to reach a steady-state solution.

## Conclusions

Vortex force is the best available option for modeling the wave contribution to storm surge in ROMS. The vortex force algorithm substantially increases the effect of wind stress on water, bringing model results closer to observations. Some adjustments are still needed (see below).

The results of all the experiments suggest making the following adjustments for ROMS when modeling wind setdown and storm surge and using the Weisberg & Zheng (2006) formulation for the drag coefficient C_d_:

1. Decrease the quadratic bottom drag coefficient RDRG2 from its default value of 3e-3 to **1e-3**. This change improves the timing of the model relative to observations.
2. Decrease vortex force by **20%**. This adjustment reduces the model's overshoot of peak setdown and surge levels.
3. If lake ice is present, increase the air-water drag coefficient *C_d_* by **10%**.

This author views the "overshoot" aspect of vortex force as a minor adjustment that should be made for this new technique after considering its use across a broad range of coastal modeling situations. I greatly appreciate the efforts of those ocean modelers who have developed the vortex force algorithm and made it available in COAWST and ROMS. The overshoot and its correction are smaller if Oey (2006) is used for the drag coefficient *C_d_*.

The wave contribution to wind setdown and storm surge is **34.7%**. If a coastal modeling study uses ROMS and does not include a wave component, that study will underestimate storm surge by about 34.7%. If there is a valid operational reason for not including waves in a forecast, such as the increased calculation time that would be required, one may consider multiplying the vertical change in the water level by 1.53 instead.
